# Information Flow Between Heart Rhythm, Repolarization, and the Diastolic Interval Series for Healthy Individuals and LQTS1 Patients

**DOI:** 10.3389/fphys.2021.611731

**Published:** 2021-06-07

**Authors:** Mateusz Ozimek, Jan J. Żebrowski, Rafał Baranowski

**Affiliations:** ^1^Cardiovascular Physics Group, Physics of Complex Systems Division, Faculty of Physics, Warsaw University of Technology, Warszawa, Poland; ^2^Institute of Cardiology, Warszawa-Anin, Poland

**Keywords:** repolarisation, heart rhythm, information flow, conditional entropy, diastolic interval

## Abstract

Using information theoretic measures, relations between heart rhythm, repolarization in the tissue of the heart, and the diastolic interval time series are analyzed. These processes are a fragment of the cardiovascular physiological network. A comparison is made between the results for 84 (42 women) healthy individuals and 65 (45 women) long QT syndrome type 1 (LQTS1) patients. Self-entropy, transfer entropy, and joint transfer entropy are calculated for the three time series and their combinations. The results for self-entropy indicate the well-known result that regularity of heart rhythm for healthy individuals is larger than that of QT interval series. The flow of information depends on the direction with the flow from the heart rhythm to QT dominating. In LQTS1 patients, however, our results indicate that information flow in the opposite direction may occur—a new result. The information flow from the heart rhythm to QT dominates, which verifies the asymmetry seen by Porta et al. in the variable tilt angle experiment. The amount of new information and self-entropy for LQTS1 patients is smaller than that for healthy individuals. However, information transfers from RR to QT and from DI to QT are larger in the case of LQTS1 patients.

## Introduction

Repolarization in the ventricles of the heart is a process allowing the muscle cells of the ventricles to regain their ability to depolarize again. Repolarization entails movement of the ions, which entered the cell during the depolarization phase of the cycle, to flow out of the cell. Specific ion channels (especially several K channels and Na/K exchangers) are responsible for this process. Repolarization may be perturbed also in the presence of heart diseases, for example, hypertrophic cardiomyopathy, coronary artery disease, and others (Dispersion of Ventricular Repolarization in Hypertrophic Cardiomyopathy) (Zareba et al., 1994; Christiansen et al., 1996; Savelieva et al., 1999).

The global or averaged electric potentials that appear on body surface electrodes (the ECG trace) are a function of the depolarization of the ventricle tissue and then of the repolarization processes in this tissue. The duration of the QRS complex is a measure of the depolarization time in the ventricles of the heart. The so-called JT interval lasting from the end of the QRS complex to the T interval is a measure of the repolarization processes in the ventricles. Ventricle repolarization interval (QT interval) duration depends importantly on the heart rate. However, the corrected QT interval (QTc) contains information on the heart rate and will not be used below for the analysis of the information flow. Another ECG interval of interest is the diastolic interval (DI)—the time between the end of the T segment and the beginning of the next QRS complex (Imam et al., 2015). During this time interval, no electrical activity occurs in the ventricles.

The interaction of the heart rate (i.e., heart rhythm as the RR interval length) and the repolarization time as measured by the QT interval are a manifestation of a single connection of the physiological network that moderates the heart cycle. The main other connections in this network are the humoral activity and the actions of the autonomic nervous system together with that of the central nervous system. Several intrinsic and extrinsic mechanisms may be linked to the interaction between the RR and QT intervals (Nollo et al., 1992; Padrini et al., 1997).

**FIGURE 1:**
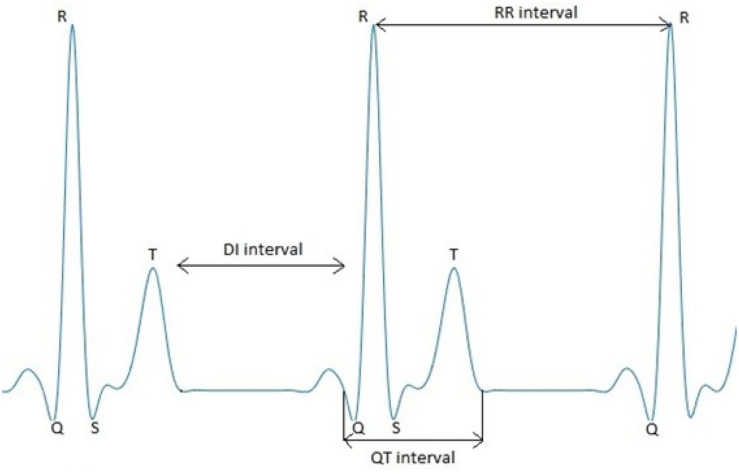
A schematic drawing of the ECG signal with marked RR interval, QT interval, and DI time series.

Our aim is to study the information flow between QT interval, RR interval, and DI time series (shown in Figure 1) using conditional entropies (Faes et al., 2015). We do not expect practical results of our research. Rather, we aim to verify on a larger group of healthy individuals than that used by Porta et al. (2017). In addition, we study the effect of the long QT syndrome type 1 (LQTS1) on the information flow.

Several authors have studied information transfer related to heart rate variability (represented by time series of RR intervals) in different contexts (Zheng et al., 2017; Javorka et al., 2018; de Abreu et al., 2020). Information flow between the QT and RR intervals was discussed by Porta et al. (2017), interval for a group of 15 healthy individuals as a function of the angle at which the tilt table is placed. They found an asymmetry between the two possible directions of the information flow between the RR intervals and the QT intervals with the information flow from the heart rhythm to the repolarization process dominating. In addition, we study information flow between these time intervals and the DI. Here, the study group of healthy individuals is larger than that analyzed by Porta et al. (2017). We are interested in the asymmetry of the information flow found by Porta et al. (2017). We also study the effect of age on the information flow between these variables and the information flow between the RR as well as the QT intervals and the DIs (Ozimek et al., 2020).

## Materials and Methods

RR intervals and the Q and T_*end*_ points were extracted using an algorithm based on Hermans et al. (2017). In the original version of the algorithm, it was used to determine the length of intervals in a 12-lead ECG recording. In our case, 3-lead recordings were available. The algorithm (Hermans et al., 2017) was used without calculating the root mean square of the signal for all leads. Each signal from each lead was treated separately, and QT intervals were determined for it. The lead for which the percentage of wrongly determined QT intervals for all the tested records was the lowest was used for the analysis. For all the individual leads, baseline deviations were subtracted from the individual ECG leads to correct for baseline wander using the algorithm in Ning et al. (2014). The filter used for baseline wander was BEADS (Ning et al., 2014), the filter order was set to 1, and the filter cut-off frequency was 0.006 cycles/sample (Ozimek et al., 2020).

The QT interval length indicates the speed at which repolarization processes in the ventricles occur. The speed of repolarization processes is a function of the heart rate. Thus, usually QT is corrected for the length of the RR interval. However, such a QTc contains information not only on the repolarization process but also on the heart rate, and so it is not suitable for studies on information flow between the heart rate and repolarization. No QT correction for heart rate was done in this paper (Ozimek et al., 2020).

To detect R waves, the Pan-Tompkins algorithm (Pan and Tompkins, 1985; Sedghamiz, 2014) was used for every individual ECG lead. The QRS onset was detected as the maximum or minimal peak of the second ECG derivative found in the window that precedes the R wave by 10–30 ms. To detect the position of the Q wave in the vicinity of ±20 ms around the R peak, the minimum of the second derivative was searched for.

To determine the T wave maximum and its end for each lead, the data were smoothed using a second order Savitzky–Golay filter (Hermans et al., 2017) in a 50 ms window. To find the T wave, the positions of the R peaks determined earlier were used. The highest or lowest value (T peak) was searched for in the range starting 150 ms after an R wave (R peak position +150 ms) ending at a point corresponding to 70% of the distance between this wave and the next R peak (R peak position +70% of the RR interval) of the smoothed signal (Hermans et al., 2017). After determining the position of the T wave, the end of the T wave was searched for. In the range from the determined T peak to a point shifted by a value equal to 30% of the distance between the surrounding R waves (T peak + 30% of the RR interval), the slope of the maximum deflection was calculated using numerical differentiation in the 10 ms window [f′(t) = (f (t+5)− f (t−5))/10]. A tangent through the point with the maximum slope was determined. The intersection of this tangent with the isoelectric line was marked as the end of the T wave (T end). The isoelectric line was locally determined as the median of amplitudes occurring from 30 ms before the onset of the QRS complex (Hermans et al., 2017).

## Data

Two databases from the THEW Project were used to provide the RR interval, QT interval, and DI series: E-HOL-03-0202-003^1^ (202 ECGs of healthy individuals) and E-HOL-03-0480-013^1^ (480 ECGs of the LQTS patients forming 4 subgroups by genotype). In this paper, we analyze only the LQTS1 patients—this is the most frequent type of the LQTS.

However, it is well known that automatic algorithms extracting the QT interval rarely work well. Below, we analyze only those ECG recordings for which our algorithm worked well. This was verified for each individual recording. We also limited the range of age of the subjects studied to 18–50 years, obtaining 84 (42 women) ECGs for healthy individuals and 65 (45 women) cases for the LQTS1 case (Ozimek et al., 2020).

## Entropy Methods

The following information theoretical methods were used (Porta et al., 2017).

The target process contains information at the present time n:

$$H_{Y}=H\,\left(Y_{n}\right).$$

Using the chain rule for information (Faes et al., 2017b), one can decompose the target information as:

$$H_{Y}=P_{Y}+N_{Y}$$

$$P_{Y}=S_{Y}+T_{X\to Y}$$

where *P*_*Y*_ is given by the mutual information due to the past of the whole network and the present of the target process, whereas *N*_*Y*_ is the new information generated in the target process as a result of the transition from the past states to the present. The mutual information *P*_*Y*_ can be decomposed into self-entropy *S*_*Y*_ and transfer entropy (TE; Faes et al., 2017b).

Self-entropy (Porta et al., 2017; Xiong et al., 2017) is a measure of the part of information that is given by the present of the target process Y that can be predicted by its own past. Self-entropy was calculated for each individual time series: RR intervals, QT intervals, and DIs:

$$S_{Y}=\sum p\,\left(y_{n}|y_{n}^{-}\right)\,l\,o\,g\,\frac{p\,\left(y_{n}|y_{n}^{-}\right)}{p\,\left(y_{n}\right)}$$

where the superscript “-” at *y*_*n*_ means the past of the target time series, vertical bars indicate conditional probability, and a bold character means that a time series was used (vector).

The information transferred from the past of the process X to the current state of the process Y is measured by the TE (Faes et al., 2015, 2017b):

$$T_{X\to Y}=\sum_{y_{n},x_{n}^{-},y_{n}^{-}\in \text{Ω}}p\,\left(y_{n},y_{n}^{-},x_{n}^{-}\right)\,l\,o\,g\,\frac{p\,\left(y_{n}|x_{n}^{-},y_{n}^{-}\right)}{p\,\left(y_{n}|y_{n}^{-}\right)}=H\,\left(Y_{n}|Y_{n}^{-}\right)-H\,\left(Y_{n}|X_{n}^{-},Y_{n}^{-}\right)$$

where *H*(|) means conditional entropy.

To assess in a simple way the asymmetry in the information flow from the signal X to the signal Y and in the opposite direction, we introduced the measure dTE:

$$d\,T\,E\,\left(X,Y\right)=T\,E_{X\to Y}-T\,E_{Y\to X}.$$

Conditional TE was used to assess the effect of the series Z on information transfer between time series X and Y; X, Y, Z = RR intervals, QT intervals, and DIs, respectively. Conditional information transfer (Faes et al., 2017b) in the form:

$$T_{X\to Y|Z}=I\,\left(Y_{n};X_{n}^{-}|Y_{n}^{-},Z_{n}^{-}\right)=H\,\left(Y_{n}|Y_{n}^{-},Z_{n}^{-}\right)-H\,\left(Y_{n}|X_{n}^{-},Y_{n}^{-},Z_{n}^{-}\right)$$

and the difference of the conditional information flow in both directions was calculated:

$$d\,T\,E\,\left(X,Y|Z\right)=T\,E_{X\to Y|Z}-T\,E_{Y\to X|Z}.$$

We introduced dTE as a simple way to show the direction of the flow. Note that this measure in some cases may be misleading as TE does not always exclusively represent the coupling strength (Faes et al., 2017b).

To estimate all conditional entropies, we used the model-free estimator based on binning (Lizier, 2014).

Information transfer decomposition–interaction information transfer (Faes et al., 2017b) $I_{X_{1},X_{2}}^{Y}=I\,\left(Y_{n};X_{1,n}^{-};X_{2,n}^{-}|Y_{n}^{-}\right)$ shows information, which is contained in the past of *X*_*1*_ and *X*_*2*_ that can be used to predict the present state of Y when *X*_*1*_ and *X*_*2*_ are taken individually. This is a measure of how the interaction of the past of *X*_*1*_ and *X*_*2*_ is transferred to the target.

$$I_{X_{1},X_{2}}^{Y}=T_{X_{1},X_{2\to Y}}-\left(T_{X_{1}\to Y}+T_{X_{2}\to Y}\right)$$

When *T*_*X*_1_,*X*_2→*Y*__ < *T*_*X*_1_→*Y*_ + *T*_*X*_2_→*Y*_ then $I_{X_{1},X_{2}}^{Y}<0$ that refers to redundant interactions contributing to the transfer of information. When *T*_*X*_1_,*X*_2→*Y*__ > *T*_*X*_1_→*Y*_ + *T*_*X*_2_→*Y*_ then $I_{X_{1},X_{2}}^{Y}>0$ that refers to synergistic interactions contributing to transfer (Faes et al., 2017b).

We normalized the data to set 0 mean and 1 standard deviation. All signals were divided into non-overlapping windows of length 600, and in all windows, we checked if the signal is mean-stationary. After the division of the signal into non-overlapping windows, the empirical mode decomposition was used to separate from signal the four last intrinsic mode functions (IMFs), to achieve mean-stationarity in a higher number of windows. The window length was chosen to maximize the number of windows for which mean-stationarity is present. We obtained slightly better results for window 400, but then we had problems with correctly calculating IMFs, so we chose the second-best result.

Because we analyzed only nighttime parts of the recordings, the average length was limited to 21,000 intervals. TE and cTE values for signals were estimated using the non-overlapping windows. Following Luca Faes in the ITS Toolbox,^2^ we set the number of quantization levels to 10, and we used embedding based on the non-uniform procedure. We checked at the beginning of our research that this value is optimal because using higher or lower values can be problematic because of calculations of conditional probability. The number of lags for each system was set to 5. During the procedure that updates the conditioning vectors, the significance test using shift surrogates was used^2^. The instantaneous effects were allowed for our analysis (Faes et al., 2015). As presented in Figure 1, the instantaneous effects go only from QT to RR.

We present results between healthy individuals and LQTS1 patients using the Kolmogorov–Smirnov test (*p* < 0.05).

## Results

### Entropy H(Y) and New Information N(Y)

**FIGURE 2:**
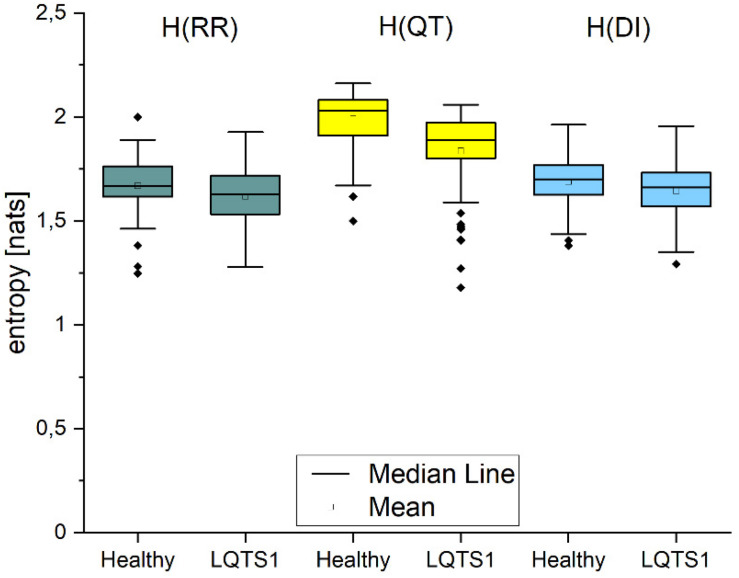
Shannon entropy H of the individual processes given by single time series: RR intervals, QT intervals, and diastolic intervals, respectively. The Kolmogorov–Smirnov test was used, *p*-values: H(RR): 0.04348, H(QT): 1.41241E-7, H(DI): 0.0998.

**FIGURE 3:**
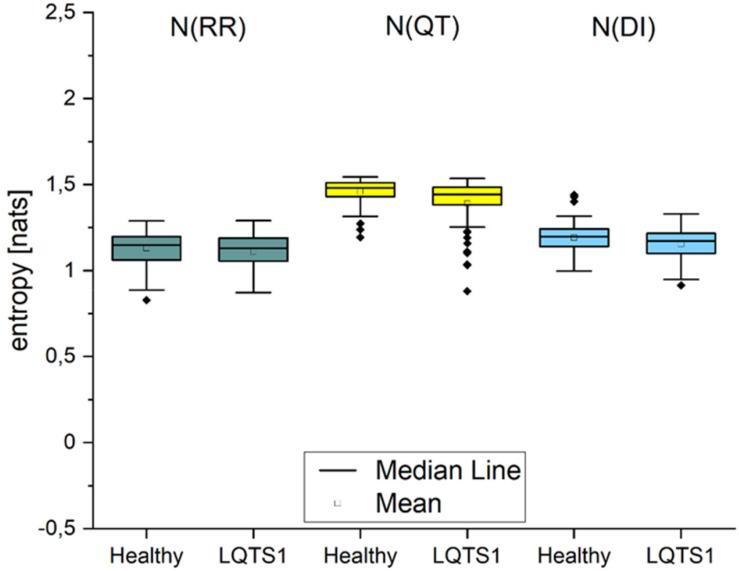
New information produced in the processes: RR intervals, QT intervals, and diastolic intervals, respectively. The Kolmogorov–Smirnov test was used, *p*-values: 0.3517 [N(RR)], 0.01124 [N(QT)], and 0.11007 [N(DI)].

The Shannon entropy H_*Y*_ calculated for the individual processes (Figure 2) shows that in all cases, it is larger for healthy individuals. We obtained statistically significant differences between healthy individuals and LQTS1 patients for RR interval, QT interval, and DI series. Figure 3 presents the boxplots of the new information Ny for all three time series. This corresponds with the amount of information that is produced at each moment in time when the past states are known (Faes et al., 2013). The information produced in all processes is larger for healthy individuals, but for N(RR) and for N(DI), we did not obtain statistically significant results.

### Self-Entropy SE

**FIGURE 4:**
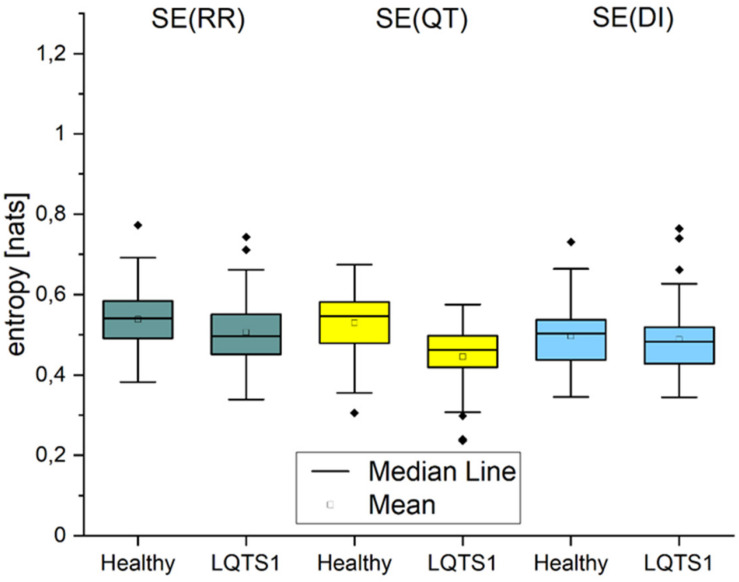
Self-entropy of the processes: RR intervals, QT intervals, and diastolic intervals, respectively. *p*-Values: SE(RR): 0.00394, SE(QT): 4.91195E-10, SE(DI): 0.06205.

The properties of the self-entropy (Figure 4) for the three types of time series were as expected: the regularity was larger for the heart rate than for the repolarization processes, and the properties of the DIs follow essentially those of heart rate variability. The larger regularity of cardiac time series for healthy individuals was reported in works studying the fluctuations in RR time series (Ivanov et al., 1999, 2001; Faes et al., 2019).

### Transfer Entropy

**FIGURE 5:**
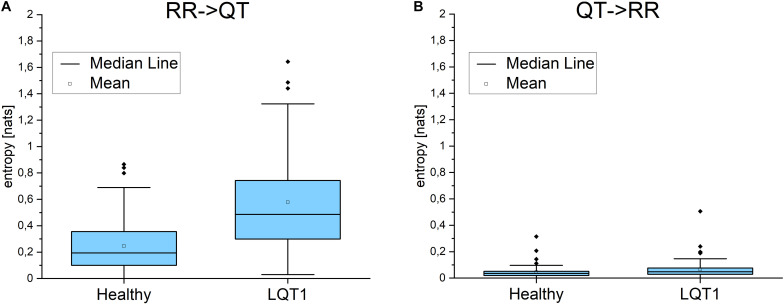
**(A)** Transfer entropy from the RR intervals to the QT intervals and in the opposite direction **(B)** for healthy individuals and for LQTS1 patients. *p*-Values: RR->QT: 8.46283E-9, QT->RR: 0.07761.

**FIGURE 6:**
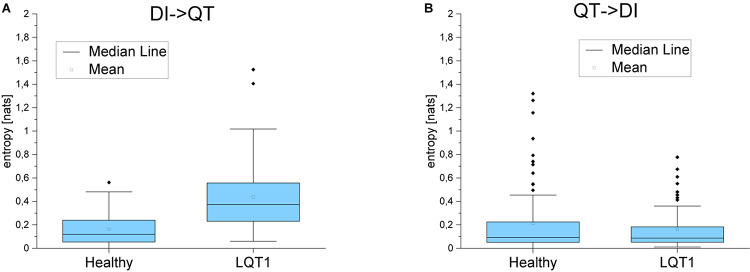
**(A)** Transfer entropy from the diastolic intervals to the QT intervals and in the opposite direction **(B)** for healthy individuals and for LQTS1 patients. *p*-Values: DI->QT: 1.60104E-9, QT->RR: 0.94574.

**FIGURE 7:**
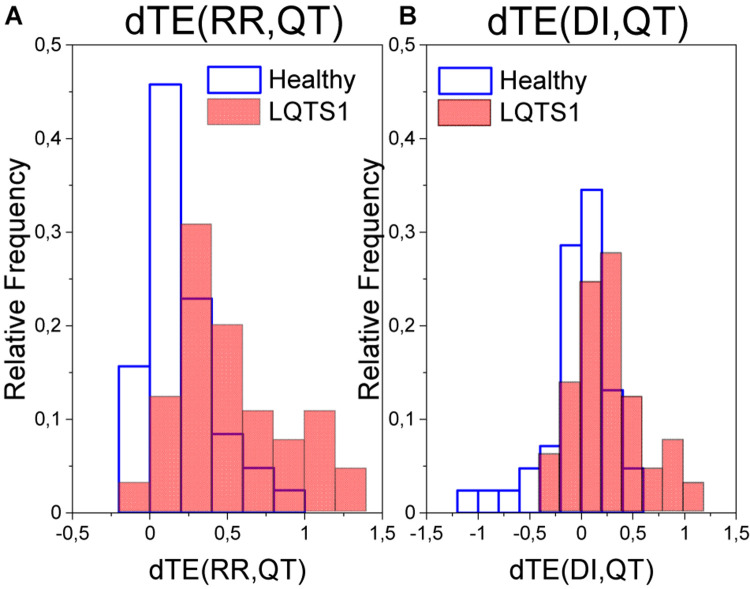
**(A)** Probability density of the difference in the transfer entropy from the RR intervals to the QT intervals and in the opposite direction for healthy individuals and for LQTS1 patients. **(B)** Probability density of the difference in the transfer entropy from the diastolic intervals to the QT intervals and in the opposite direction for healthy individuals and for LQTS1 patients.

**FIGURE 8:**
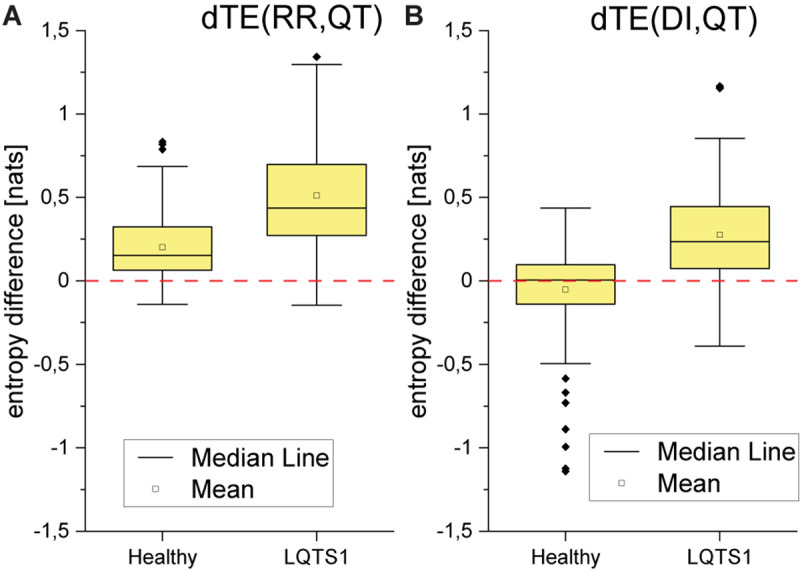
**(A)** Boxplot of the difference between TE in both directions for the heart rhythm (RR intervals) and the repolarization (QT intervals). **(B)** Boxplot of the difference between TE in both directions for the diastolic interval series (diastolic intervals) and the repolarization (QT intervals). *p*-Values: dTE(RR,QT): 8.57094E-9; dTE(DI,QT): 7.55201E-8.

For the majority of healthy individuals, TE from RR to QT was between 0.1 and 0.4. On average, TE in the direction from QT to RR was significantly smaller for LQTS1 patients and for healthy individuals, most often with TE less than 0.05 (Figures 5A, 5B). Similarly as Porta et al. (2017), we observed an asymmetry in the information flow between heart rhythm and the QT time series for LQTS patients: the parameter *dTE* = *TE*(*RR*→*QT*)−*TE*(*QT*→*RR*) for a majority of the patients was positive. The results for the information flow between DI and RR are similar, but TE values of *QT*→*DI* flow are higher (Figure 6). However, rather surprisingly, for LQTS1 patients, we observe higher values of dTE(RR, QT) than for healthy individuals: this can be seen in the histogram of dTE(RR, QT) (see Figure 7A below). On average, the information flow from the repolarization process to the heart rhythm was much smaller (Figure 8A). The flow itself was also small: the majority of the TE values were less than 0.05. The asymmetry was present in our results: the expectation value of the difference dTE(RR, QT) was larger for the LQTS1 group and remarkably close to 0 for the healthy group. The results for DI follow the results for RR intervals (Figures 7B, 8B). We did not observe statistically significant difference between healthy individuals and LQTS1 patients in the case of *QT*→*DI* flow.

**FIGURE 9:**
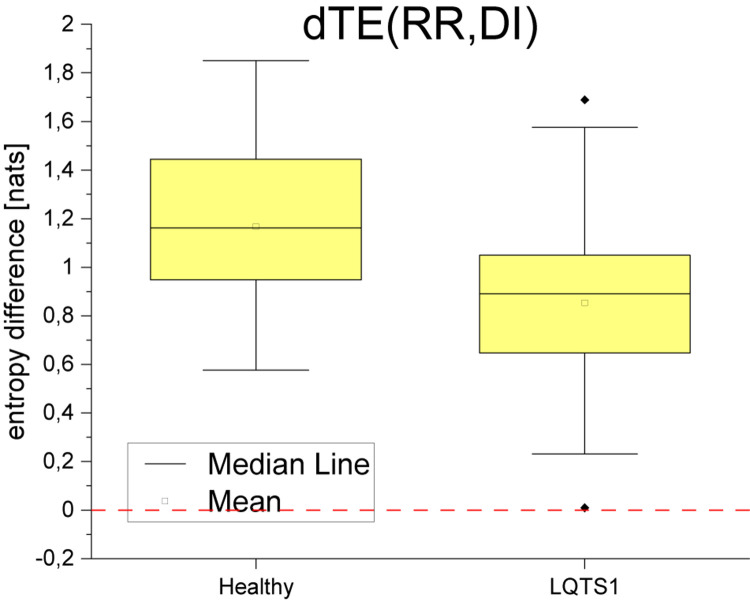
Boxplot difference between TE in both directions for the diastolic intervals and heart rhythm (RR intervals). *p*-Value: 2.74318E-6.

For the LQTS1 patient group, the flow is very asymmetric, dTE(RR,DI) is larger than zero with a significant dispersion (Figure 9). Information flow between DI and QT is practically opposite to the behavior of the flow between DI and heart rhythm. In all cases, the information flow from the DI series to the heart rhythm dominates.

### Conditional TE

Conditional information transfer provides information on how the information flow between two variables depends on the time evolution of a third variable. We present the differences dTE between the respective conditional entropies calculated in both directions.

**FIGURE 10:**
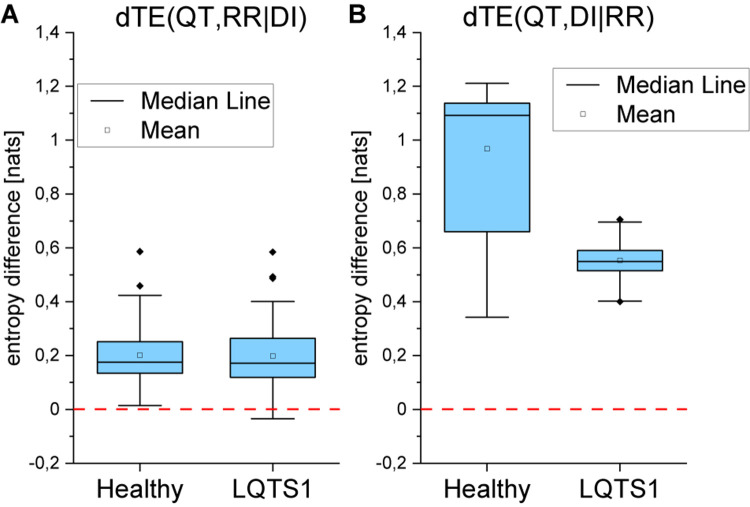
**(A)** Conditional transfer entropy for the information flow between repolarization (QT) and heart rhythm (RR intervals) given the diastolic intervals (DIs) for healthy individuals and for LQTS1 patients. **(B)** Conditional transfer entropy for the information flow between repolarization (QT) and the DIs given the heart rhythm (RR intervals) for healthy individuals and for LQTS1 patients. p-Values: dTE(QT,RR| DI): 0.52989, dTE(QT,DI| RR): 0.00205.

Figure 10B shows that a strong asymmetry between the flow from the QT interval and the DI series occurs. The flow from QT to DI dominates, and the asymmetry is smaller for the LQTS1 group. We obtained a different result for dTE(QT, RR| DI) that is presented in Figure 10A. The flow from the QT interval to the heart rhythm (RR intervals) is small. In Figure 10B, it can be seen that the information flow given the heart rhythm from the repolarization processes (QT intervals) to the DI for healthy individuals dominates over the flow in the opposite direction. On the other hand, for LQTS1 patients, this asymmetry is much smaller so that the flow from the DIs to the QT time series given the heart rhythm is much less pronounced. At the same time (Figure 10A), the conditional information flow from the repolarization processes in the ventricles to the heart rhythm given the DI series is about the same in both healthy individuals and LQTS1 patients. The conditional dTE is positive so that the flow from the repolarization processes (QT intervals) to the heart rhythm dominates although much less than for the results shown in Figure 10B.

**FIGURE 11:**
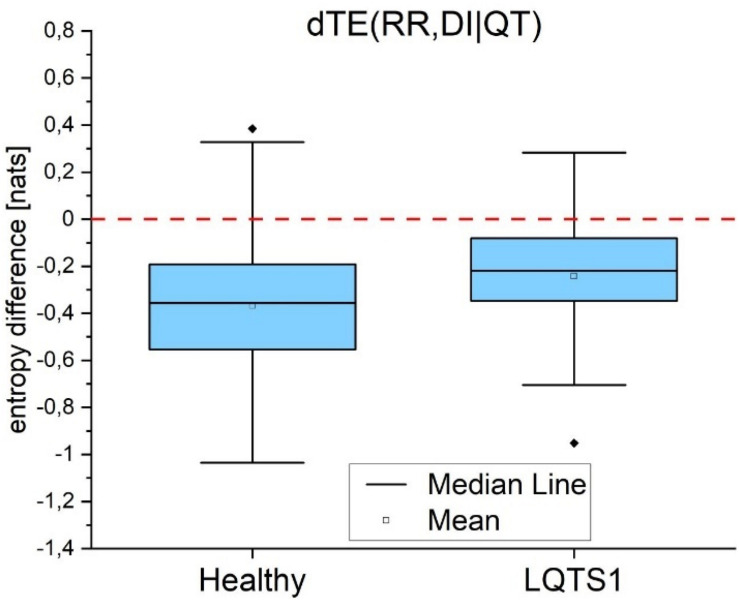
Conditional transfer entropy difference for the information flow between heart rhythm (RR intervals) and the diastolic intervals given the repolarization (QT intervals) for healthy individuals and for LQTS1 patients. The Kolmogorov–Smirnov test was used, *p*-value = 0.002.

Conditional transfer entropy difference dTE(RR,DI| QT) is negative, and the modulus of this difference is on average larger for healthy individuals than for LQTS1 patients (Figure 11).

### Redundancy and Synergy (Faes et al., 2017b)

Using the theory of interaction information decomposition (Faes et al., 2017a), one can decompose the information that a vector of values *X* = {*X*_1_,*X*_2_} provides about system *Y* into terms, which are connected with information contributed individually by *X*_1_,*X*_2_ and jointly.

Interaction information for the time series studied here may be written as:

$$I_{R\,R,D\,I}^{Q\,T}=T_{R\,R,D\,I\to Q\,T}-\left(T_{R\,R\to Q\,T}+T_{D\,I\to Q\,T}\right)$$

$$I_{R\,R,Q\,T}^{D\,I}=T_{R\,R,Q\,T\to D\,I}-\left(T_{R\,R\to D\,I}+T_{Q\,T\to D\,I}\right)$$

$$I_{Q\,T,D\,I}^{R\,R}=T_{Q\,T,D\,I\to R\,R}-\left(T_{Q\,T\to R\,R}+T_{D\,I\to R\,R}\right).$$

**FIGURE 12:**
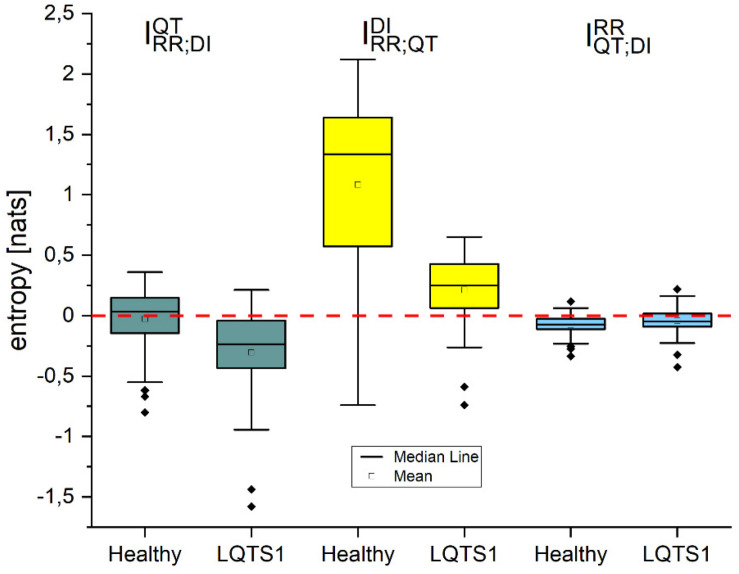
Information interaction for healthy individuals and for LQTS1 patients for three combinations of the variables: RR intervals, QT intervals, and diastolic intervals. The Kolmogorov–Smirnov test was used, *p*-values: $I_{R\,R;D\,I}^{Q\,T}$: 2.105E-13, $I_{R\,R;Q\,T}^{D\,I}$: <  10^−3^, $I_{Q\,T;D\,I}^{R\,R}$: <  10^−3^.

Figure 12 shows that for $I_{Q\,T,D\,I}^{R\,R}$ and $I_{R\,R,D\,I}^{Q\,T}$, one can observe rather redundant interactions, which are stronger for the healthy individual group for $I_{R\,R,D\,I}^{Q\,T}$ and for the LQTS1 patient group for $I_{Q\,T,D\,I}^{R\,R}$. In the case of $I_{R\,R,Q\,T}^{D\,I}$, one can observe synergetic interactions. For the average result for $I_{R\,R,Q\,T}^{D\,I}$ in the LQTS1 group, synergy is the lower. There is also a remarkable group of cases for which we observe redundancy.

## Discussion and Conclusion

A part of the cardiovascular physiological network (the relation between heart rhythm, the DI series, and the uncorrected QT time series) was analyzed for two groups: healthy individuals and LQTS1 patients.

For single time series, calculations of new information show that both for healthy individuals and for LQTS1 patients the heart rhythm as well as the DI series have similar properties, and that the main difference between the two groups is seen in the repolarization process. For the QT intervals, we obtained a larger new information N(Y) for healthy individuals. This can be associated with a higher complexity of the process dynamics (Zanetti et al., 2019).

For self-entropy estimations, we observed that QT regularity in healthy individuals is larger than that in LQTS1 patients and heart rate regularity for healthy individuals is on average larger and more complex than that for LQTS1 patients. This difference in regularity may result from larger vagal reactivity for LQTS1 patients (Bari et al., 2014) (Cairo et al., 2019). However, removing the trend using the EMD method may have some influence on this result. We expect the opposite—greater QT regularity in the group of LQTS1 patients (Seethala et al., 2015; DeMaria et al., 2020). Moreover, for a regularity parameter, such as SE, the sequential order of data is very important in contrast to variability measures (Pincus and Goldberger, 1994). For LQTS patients and for healthy individuals, DI regularity shows no statistically significant differences between groups.

In the case of RR and QT intervals analysis, calculations of TE confirm well-known results (Porta et al., 2017). We observe an asymmetry in the information flow between heart rhythm and the QT time series.

**FIGURE 13:**
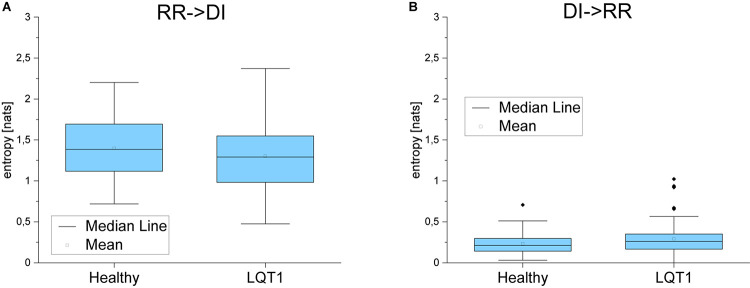
**(A)** Transfer entropy from the RR intervals to the diastolic intervals and in the opposite direction **(B)** for healthy individuals and for LQTS1 patients. *p*-Values: RR->DI: 0.3764, DI->RR: 0.20194.

The behavior of the DI series is similar to the behavior of heart rate variability that is also to be expected. DI is a function of other factors than the RR interval. The RR interval is moderated by autonomic regulation depending on the requirements for body function, whereas DI is more related to internal processes occurring inside the heart. Hence, the difference seen in Figure 13, for example. This indicates that both for healthy individuals and for LQTS1 patients the heart rhythm as well as the DI series have similar properties, and that the main difference between the two groups is seen in the repolarization process. On average, for LQTS1 patients, more information flows between heart rhythm and the QT time series than it does for healthy individuals—in both directions. For the LQTS1 group (except for a small group of outliers), the information from heart rhythm to QT dominates (dTE > 0). For healthy individuals, the distribution of dTE(RR,QT) has lower dispersion. The flow from QT to heart rhythm is a new result, but TE in the direction from QT to RR is much slower than that from RR to QT.

Conditional TE shows that the flow between the QT interval and the DI series when RR interval is given is asymmetric. The flow from QT to DI is much larger than in the opposite direction. However, the flow between QT and RR time series when DI is known shows no statistically significant difference between healthy individuals and LQTS1 patients. For healthy individuals, the conditional information flow from the DIs to the heart rhythm dominates—given the repolarization processes. This effect is larger for healthy individuals than for LQTS1 patients indicating that the LQTS pathology decreases the adaptability of the physiological network decreasing the interaction between the heart rhythm and the DIs. For interaction information decomposition, we observe in most cases redundant interactions. For $I_{R\,R,Q\,T}^{D\,I}$, the combination of RR intervals and QT intervals gives additional information on the DIs, which is not available from either time series alone. In this case, synergy is observed. However, it should also be remembered that histogram-based methods of estimation of probabilities have the problem of a large bias (Panzeri et al., 2007; Faes and Porta, 2014). It could affect the results because it is not compensated while many entropy terms are summed together. The results for $I_{R\,R,Q\,T}^{D\,I}$ could be affected the most—TE values from the QT intervals to the DIs are characterized by many outliers, and joint information transfer from RR and QT to DI is also high; however, these values can be inflated by histogram-based estimator.

## Data Availability Statement

The data analyzed in this study is subject to the following licenses/restrictions: Both data sets belong to the THEW Project (http://thew-project.org/databases.htm) available upon registration. Requests to access these datasets should be directed to http://thew-project.org/databases.htm.

## Ethics Statement

Ethical review and approval was not required for the study on human participants in accordance with the local legislation and institutional requirements. The patients/participants provided their written informed consent to participate in this study.

## Author Contributions

MO: all calculations, all figures, and basic structure of the manuscript. JZ: general supervision, enhancement of the language, and basic structure of the manuscript. RB: initiator of research and medical science supervisor. All authors contributed to the article and approved the submitted version.

## Conflict of Interest

The authors declare that the research was conducted in the absence of any commercial or financial relationships that could be construed as a potential conflict of interest.

## Footnotes

1 THEW. Available at: http://thew-project.org/Database/E-HOL-03-0202-003.html

2 ITS Toolbox, available on: http://www.lucafaes.net/its.html
